# Initial results of the meteorological data from the first 325 sols of the Tianwen-1 mission

**DOI:** 10.1038/s41598-023-30513-2

**Published:** 2023-02-27

**Authors:** Chunsheng Jiang, Yu Jiang, Hengnian Li, Sen Du

**Affiliations:** 1 State Key Laboratory of Astronautic Dynamics, Xi’an, China; 2 Xi’an Satellite Control Center, Xi’an, China; 3 Polytechnic University of Catalonia, Barcelona, Spain

**Keywords:** Atmospheric science, Climate change, Astronomy and planetary science

## Abstract

As the Zhurong rover landed on the surface of Mars in 2021, it began a months-long collection of Mars data. Equipped with highly sensitive sensors, Zhurong is capable of being a meteorological station at the surface of Mars. The Mars Climate Station, one of the onboard sensors with high sensitivity, helps the Tianwen-1 lander to collect meteorological data at the Martian surface, via which the air temperature, atmospheric pressure, wind speed and direction are measured. In this paper, we present results of surface pressure, air temperature and wind data from the Mars Climate Station at Zhurong’s landing site. The data is collected in 176 solar days out of the entire rover’s mission time, 325 solar days. We use a trigonometric function to fit the relationship between the solar longitude (Ls) and the pressure, after which we compare the results with those of Viking I. Our analysis of the temperature shows that seasonal evolution is similar to the patterns concluded in previous Mars missions at different landing sites. We discover that wind speed appears the maximum in early summer near Zhurong’s landing site, and analyze the occurrence of dust storms by combining the data of wind and temperature. Our results provide some evidence of the seasonal changes in meteorological pattern at Tianwen-1’s landing site, south of Utopia Planitia. With the mission ongoing further, more results are expected in the future.

## Introduction

In the solar system, Many features of Mars are similar to those of Earth, such as wind, clouds, and a rotation period of about 24 h. Mars is about half the size of Earth which means that Mars is the second smallest planet in the solar system behind Mercury. The surface of Mars has many marks left by years of wind and sunshine. These marks preserve a record of Mars' formation and provide insight into how terrestrial planets developed. Mars brings an opportunity in front of us to find proper answers to mysteries including the existence of extraterrestrial life, the formation of the solar system, and the possibility of human habitation. The earliest Mars surface exploration dates back 40 years to the twin Viking missions^1,2^, and thereafter, several missions aiming to unravel the modern near-surface climate of Mars have been performed. Mars Pathfinder with the Atmosphere Structure Instrument/Meteorology package operated 84 sols to study the variability of the Martian atmosphere at Ares Valley. However, the temperature the rover suffered was much lower than its design limit. As a result, it did not reach a conclusion about the temperature change^3^. Sojourner, part of the Mars Pathfinder mission, was the first wheeled vehicle to rove on a planet other than Earth^4^. The Mars Exploration Rover mission (MER) has two rovers, Spirit and Opportunity, whose meteorology science goal was to provide high vertical resolution temperature profiles in the boundary layer, to measure dust and ice aerosol optical depth, and to determine aerosol properties from imaging. As Spirit’s measurement data spanned over 2000 sols and Opportunity’s covered more than 4000 sols, sufficient data on atmospheric opacity were obtained, and the air temperature of Mars surface was measured for the first time^5^. In 2007, NASA launched the Phoenix which was fixed in one place and stayed for a long time working in the North Pole region of Mars. Phoenix carried a meteorological station to measure air temperature, pressure, and wind at different heights. It was the first Mars polar exploration on climate evolution in human history^6,7^. The Mars Science Laboratory (MSL) Curiosity, nuclear powered, carried a more complex and capable scientific payload. A comprehensive diurnal and seasonal surface wind characterization was presented for the location of MSL in Gale Crater, and the characterization of Martian surface winds as a function of time of day and season at one location was presented which increased the knowledge of Mars surface conditions and assist in planning for future unmanned and manned missions^8^. The 2020 Mars lander InSight (Interior Exploration using Seismic Investigations, Geodesy and Heat Transport) measured Mars’s atmosphere with unprecedented continuity, accuracy and sampling frequency. Detailed analysis on large-scale atmospheric phenomena, diurnal and subdiurnal variability and turbulence studies were carried out^9^. The results showed that during the 220 sols of InSight’s work, the pressure sensor had detected pressure drops by about a thousand times corresponding to convective vortices. The Mars 2020 Mission Perseverance was designed to better understand the geology of the Red Planet. A new result showed that the distribution of observed pressure excursions for the vortex encounters satisfied a power-law fit and the hour-by-hour encounter rate varied throughout the sol with a peak near mid-day^10^.

In July 2020, China executed its first Mars mission, Tianwen-1 probe whose scientific objectives include^11^: (1) to characterize the Martian morphology and geological structure, (2) to study surface soil characterization and water–ice distribution on Mars, (3) to characterize the composition of Martian surface, (4) to explore Martian ionosphere, surface climate and environmental characteristics, and (5) to map electromagnetic and gravitational fields, and internal structure of Mars. Tianwen-1 comprises two parts: the orbiter and the rover. The orbiter is responsible for small-scale, or global, observation of Mars, revealing clues about large mountains and grand canyons, while the rover mainly focuses on regional area observations. Additionally, the orbiter provides a relay communication link to the rover, while performing its own scientific observations. After about three-month circling around Mars at a parking orbit, the rover, known as Zhurong, was released in May 2021 touching down on Utopia Planitia, close to the boundary between Martian northern lowlands and southern highlands, de facto in the northern lowlands of Mars (109.925° E, 25.066° N)^12^. Choosing the landing site is a strong guarantee for follow-up work (“Methods”).

**Figure 1:**
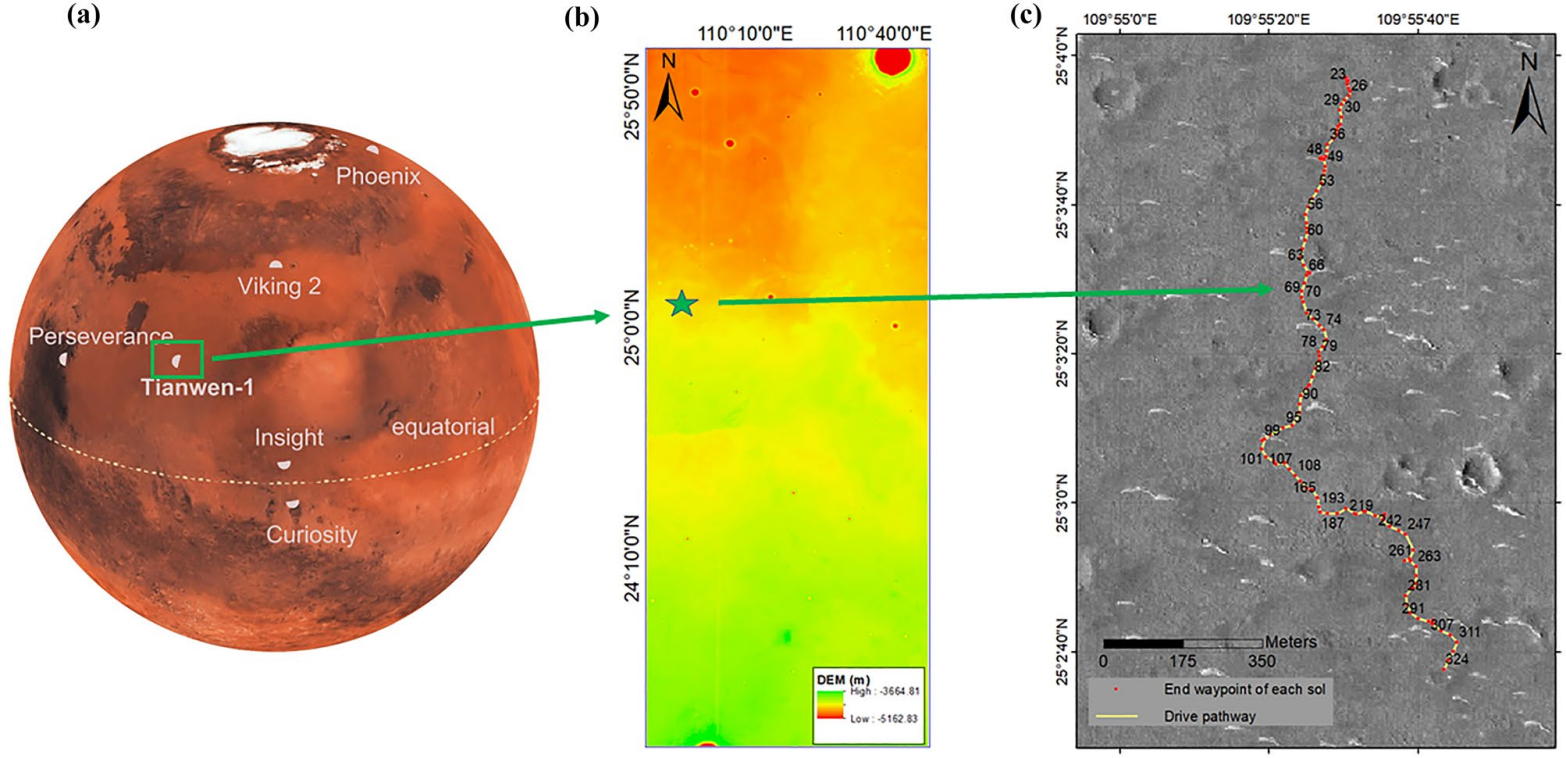
Location of the Tianwen-1’s landing site on Mars, (**a**) along with other landers and rovers operated at the surface of Mars. The south of Utopia Planitia is a place that has not been visited by previous missions. (**b**) Digital elevation map of Utopia Planitia taken by Tianwen-1’s high-resolution imaging camera. (**c**) The drive pathway of the Zhurong rover during its first 325 sols. The red points represent the end waypoint on each sol.

Utopia Planitia is believed to emerge from a large impact^13^ and it is the largest basin in the northern hemisphere of Mars. Figure 1a compares the landing site with those of previous missions. Figure 1b shows the digital elevation map of Utopia Planitia taken by Tianwen-1’s high-resolution imaging camera. The star icon represents the location of Tianwen-1’s landing site. Figure 1c shows the pathway of the Zhurong rover. Having attracted all eyes on the earth, Zhurong now joins several other active Mars missions, and becomes the third alive rover around the world on the Martian surface.

As evidenced from previous missions, Mars has daily weather variations^14,15^. The greatest source of climate variability on Mars is associated with major dust storms, owing to the large impact of dust loading on the thermal state of the thin Martian atmosphere^16,17^. Our knowledge of near-surface meteorology is mostly gleaned from the few surface missions to date, which has largely provided bulk variables (temperature, pressure, horizontal wind, etc.) at a single height above the surface^18^. For Earth atmospheric models, parameterizations of these processes are based on numerous rigorous field observations. For Mars, environment measurements are limited. However, the environmental parameters including atmospheric temperature, pressure and wind can be combined to analyze some phenomena on Mars.

The Mars Climate Station (MCS), one of the principal scientific payloads of Tianwen-1, consists of four measurement sensors which collect the Martian surface environment characteristics such as temperature, pressure, wind, and sound. MCS is a good prototype for a future network of geophysical-meteorological stations at the surface of Mars. This study does not provide an exhaustive description of Tianwen-1’s instrumentation. More details of the MCS are introduced in literature^19^. We are aimed to characterize Martian climate and to provide advance predictions of the meteorological phenomena and seasonal patterns and contribute to more desirable human exploration of Mars in the future. It seems that many observed examples of surface changes can be explained by combined factors of wind, temperature, and pressure^20^. Further research on changes in the environment contributors along with concurrent measurement help to build quantitative models under Martian conditions. This is fundamental to understanding the climate processes on Mars.

In this work, we will outline observed and interpreted surface activities occurring within the Mars environment. We analyze the seasonal changes on the Martian surface and present initial results by Zhurong’s meteorological sampling data of its first 325 sols approximately.

## Results

### Surface pressure on Mars

Pressure is a key parameter in characterizing the properties of the Martian surface formation. High level of precision and sampling of the pressure sensor can help to provide a more complete view of the statistics of dust devil events^21^, the seasonal behavior of thermal tides^22^, and the variety of planetary-scale waves^23^. Prior to Tianwen-1, surface pressure was estimated by a remote method based on ground measurement^24^ or spacecraft^25^. Also, previous landings on Mars provided high-quality and long-span in-situ measurement data. These data help researchers to understand Mars to a large extent.

MCS recorded data of 176 sols during its first 325-sol-journey. In the first 91 sols, MCS worked in a traditional mode during which the equipment status was checked. To ensure the success of the scientific mission, the validity of the telemetry data was analyzed at the beginning. After 91 sols, MCS worked in a maneuvering mode. In the traditional mode, MCS recorded data for about 5 min, while during the maneuvering mode, it recorded for about 40 min. Though the data were not recorded continuously for the mission time, the climate data were the first in-situ measurements in the south of Utopia Planitia.

**Figure 2:**
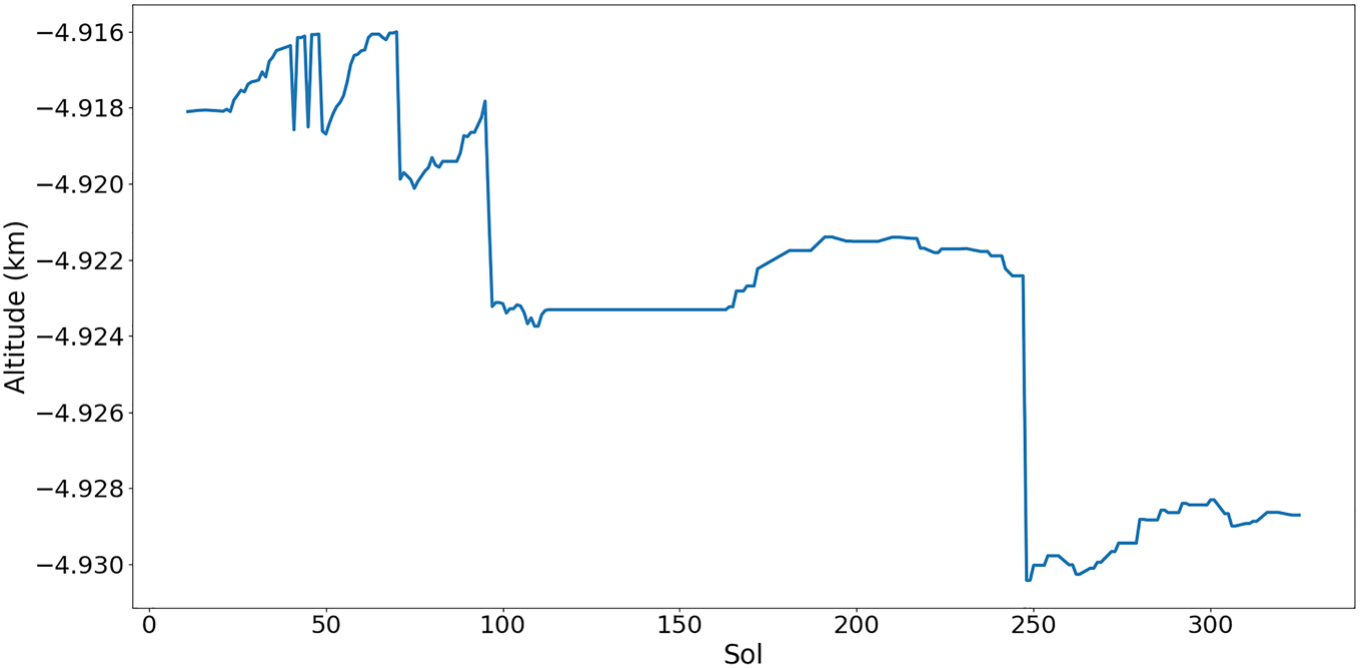
The altitude of the Zhurong rover changes during its first 325 sols mission time.

Figure 2 shows the altitude of the Zhurong rover changes with sols. The places of the sudden drop in the curve very likely correspond to the existence of crater. It is not the main problem discussed in this paper, but still meaningful for further studies on Mars topography.

**Figure 3:**
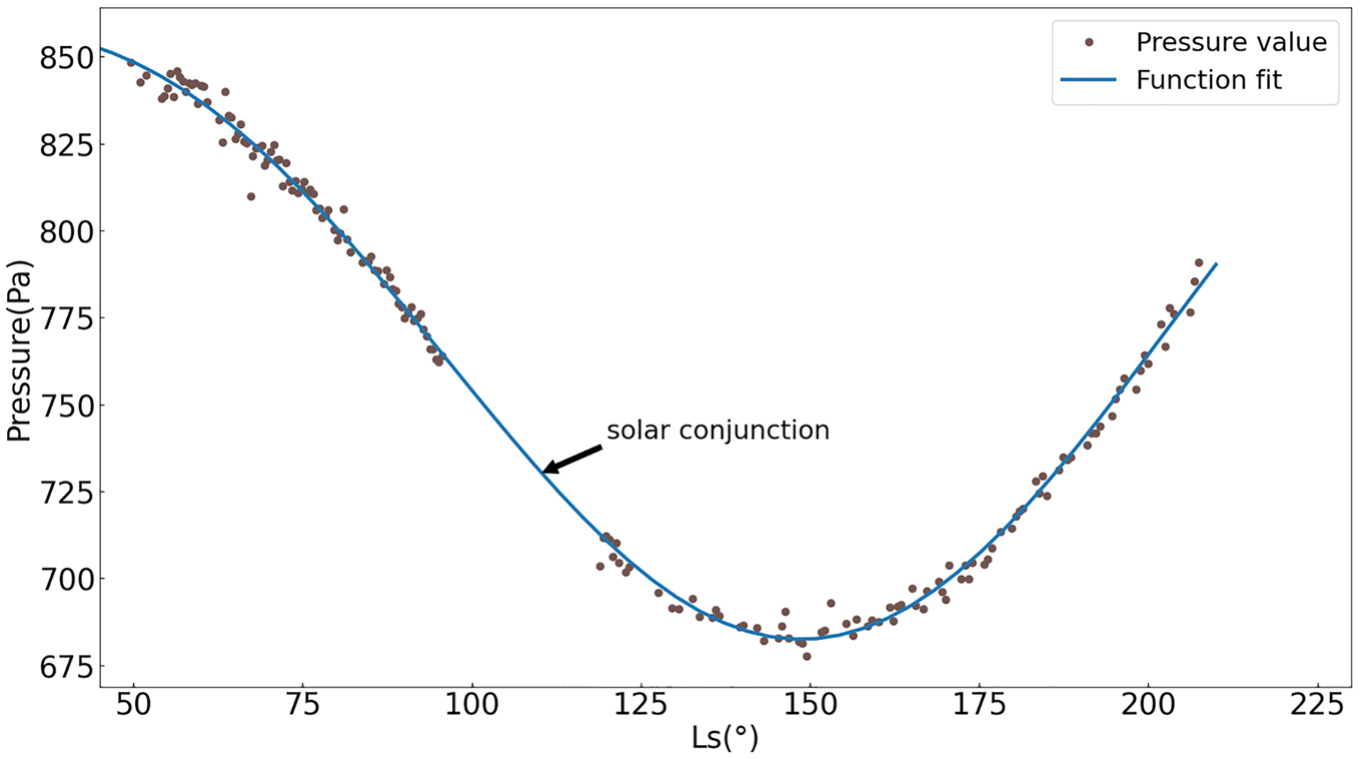
Pressure fit using a trigonometric function. The figure shows an obvious trend with the season. Each point is an average value in one sol to prevent bias from the fact that there may be a significantly different number of measurements taken at different times of a sol. The Zhurong rover and the Tianwen-1 orbiter work in a safe mode and stop collecting data during the solar conjunction, and this leads to a blank of 50 sols. Blue curve is the one fitted by the data of about 12 months. Real pressure points distribute just nearby the line.

The pressure values recorded during the mission are shown in Fig. 3. The Mars–Sun angle spans from 50° to about 208°. The data per sol is the average value of the data recorded in one sol. The maximum value is 848.25 Pa which appears at the beginning of the date. The minimum value is 677.70 Pa corresponding to the Sol 213 and Ls = 149.5°. The evolution of the mean surface pressure at the Zhurong landing site manifests a similar trend as NASA’s InSight mission^9^. An empirical expression for diurnal mean surface pressure is used to fit the pressure points and a result is obtained in Fig. 2. The method proposed by Withers^26^ is to predict the surface pressure in support of the landing of the Mars Science Laboratory. The form of the function is $p = p_{0} \exp \left( { - \left( {z - z_{0} } \right)/H_{0} } \right)\left[ {1 + s_{1} \sin \left( {L_{s} } \right) + c_{1} \cos \left( {L_{s} } \right) + s_{2} \sin \left( {2L_{s} } \right) + c_{2} \cos \left( {2L_{s} } \right)} \right]$. The coefficients of the function for the best fit are *p*_0_ = 763.8 Pa, *z*_0_ =  − 4.35, *H*_0_ = 11 km, *s*_1_ =  − 0.060, *c*_1_ = 0.083, *s*_2_ = 0.042, *c*_2_ =  − 0.027. The coefficients are slightly different from Withers’. The reason is that the data used by Withers come from Viking I whose landing site, compared with Tianwen-1’s, is on the other side of Mars. As the locations and the meteorological conditions are different, the coefficients are different as well.

**Figure 4:**
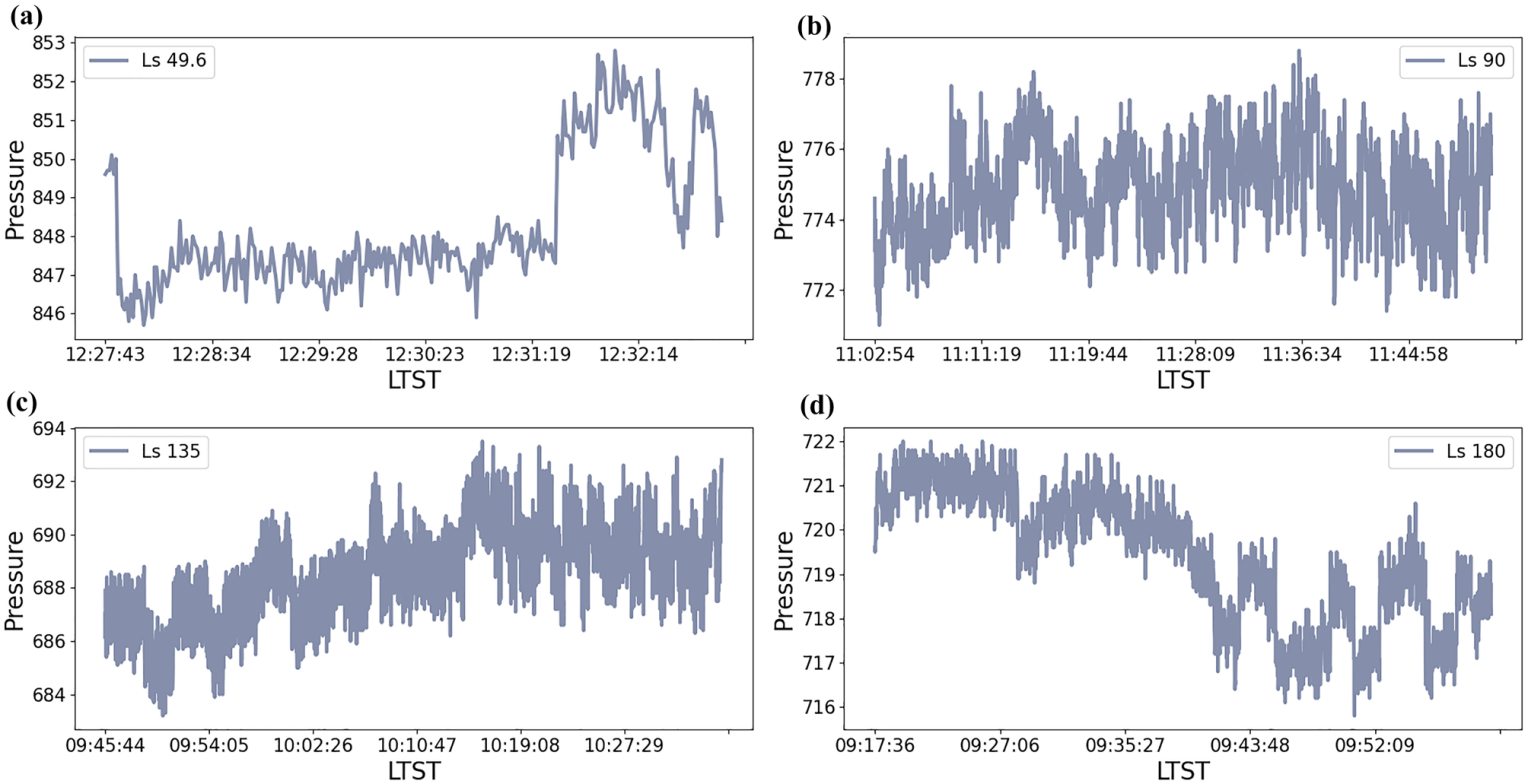
The pressure bumps occur in one sol at different Ls. For (**a**), where Ls = 49.6°, since the Zhurong rover just landed on the surface of Mars, it works with a traditional mode in order to acclimate to the Martian environment and test the sensors capabilities. For that, only 5-min information is recorded. For (**b**–**d**), the rover works with a maneuver mode and the pressures are recorded about 40 min.

Furthermore, the data demonstrate that pressure is highly variable from one sol to another and undergoes significant seasonal variability. Also, the amplitude changes of pressure during one sol at different Ls are compared in Fig. 4.

It can be seen in each subfigure that the surface pressure range of Mars varies widely over the course of one sol. The pressure observations see a few similar kinds of pressure drops attributed to diurnal thermal vortices^22,27^ and the motion of baroclinic eddies^23^. Pressure jumps are particularly obvious in Fig. 4a,d which suggest bores might occur^9^. Similar phenomena have been detected by e.g., Pathfinder mission^28,29^. Research on the Perseverance connects the diurnal perturbations with the thermal tide and the unknown global CO_2_ cycle^30^. We cannot reach a clear conclusion on the diurnal pressure jump but we can offer some evidence on the phenomena for further analysis. In different subfigures, however, the pressures show a slight change in different Martian seasons. Pressure in different seasons recorded by Zhurong is similar to that of the Perseverance but about 40 Pa lower than that of the InSight^9^. It is probably due to the fact that Zhurong and the Perseverance have similar latitudes while the InSight lands on a place near the equator. There is a correlation between the latitude and pressure range.

### Air temperature on Mars

Air temperature is a key value in both weather and climate sciences. Several missions have measured the near-surface temperature of the Martian atmosphere. The Viking landers, Mars Pathfinder, Phoenix, Curiosity and InSight all carried thermometers^31^. Pressure is a probe of both local and global phenomena, while temperature is a probe of the local atmosphere and strongly depends on latitude. Temperature is impacted by many factors like dust storms, gravity waves, and diurnal tides. Zhurong’s high-sensitivity temperature measurements are valuable references to study these phenomena in mid-latitudes.

**Figure 5:**
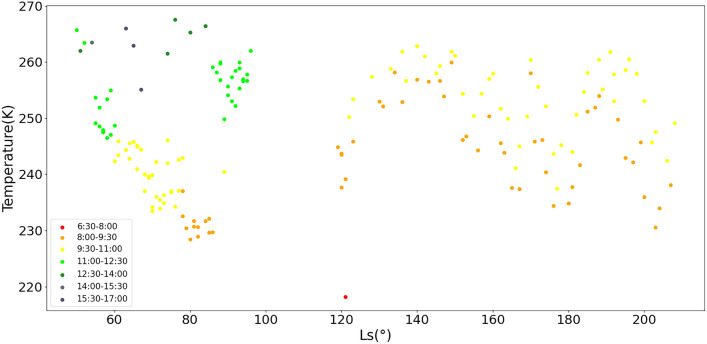
Temperature changes during mission time. Different colors correspond to different time slots.

Figure 5 describes that the temperature changes with the sol. Larger seasonal temperature amplitudes are observed by landers closer to the poles such as Viking 2^32^. The recorded starting time and duration for temperature by MCS vary nearly every sol. According to Spiga’s results on InSight^14^, a ~ 60 K temperature variation exists between day and night on Mars. The lowest temperature in one sol appears at about local true solar time (LTST) 5:00 and the highest appears at about 15:00. During this time, the temperature rises with time linearly. As the temperatures monitored by Zhurong are all collected within this period, we reasonably infer that about 6 K increases in 1 h. The highest average temperature recorded during the mission by Zhurong is about 268 K and the lowest is about 218 K. From Fig. 5, it can be seen that the lowest temperature also the only sample appears at LTST 6:30–8:00, which basically reflects the lowest temperature in the early summer in the south of Utopia Planitia. Most of the recorded time mainly lies in two slots, the first is between the LTST 8:00 and 9:30, and the second is between LTST 9:30 and 11:00. As a result, these data (yellow and orange points in Fig. 5) are used to analyze the change pattern of temperature. The temperature decreases gradually before the solar conjunction, after which it increases. It can be inferred that the lowest temperature comes at about Ls = 90° corresponding to the northern summer solstice. According to the results of 1600 sols collected by the Curiosity rover^14^, the temperature peaked around Ls = 180°. However, in accordance with the results of the Zhurong rover, the temperatures fluctuate between Ls = 160° and Ls = 200° and drop at Ls = 160° and Ls = 180°. Utopia is one of the preferred regions for the northern dust storm sequences, and its sequences travel very long distances from the north to the south^33^. Therefore, the occurrence of temperature bumps is likely due to the fact that dust storms more likely happen at that time^34^.

**Figure 6:**
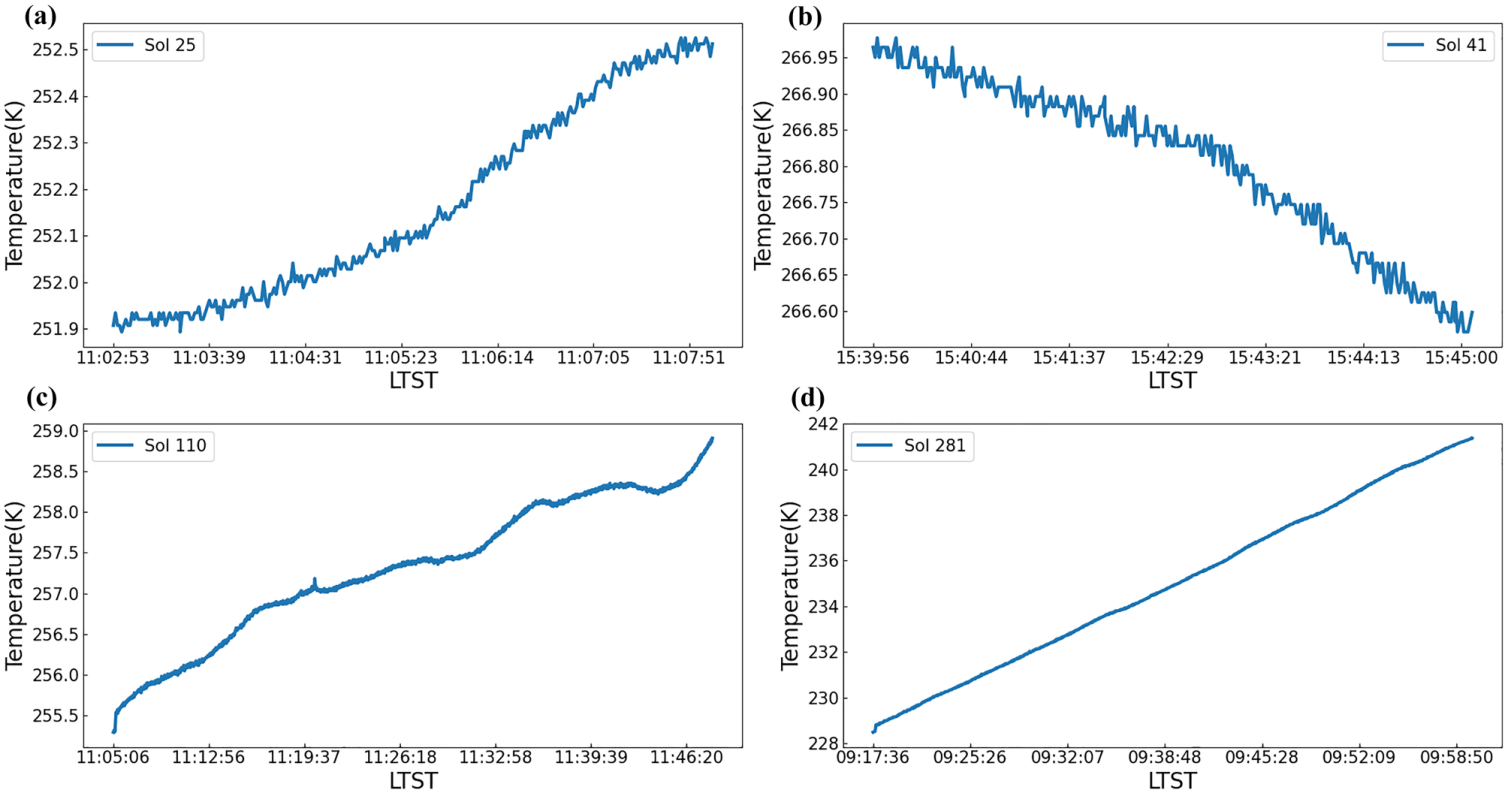
Temperatures recorded in different sols. Due to the limitations of flight control conditions and multiple-sensor coordination, temperature measurement information is not available every day. Each line in the figure represents the change in temperature during one sol. It can be seen from the subfigures that the temperature is recorded in less than 1 h, more exactly, about 5 min during the traditional mode and about 40 min during the maneuver mode. (**a**, **b**) are MCS working in traditional mode. (**c**, **d**) are MCS working in maneuvering mode.

MCS works in different modes at different time slots. Figure 6 illustrates how the temperature recorded changes in one sol. We select four sols to illustrate the changing trend of temperatures. In the traditional mode, temperature spans less than 1 K in 5 min but the changing trend is obvious. In the maneuvering mode, temperature spans across different scales. The variation range of 9:00 time slot is wider than that of the other. Another notable phenomenon is that the measurement of air temperature is affected by different factors. When winds and convection are strong, the advective heat transferring to the sensors dominates. On the contrary, when winds are low, radiative effects play a more significant role. These perturbations may reach as high as 10–15 K. As a result, the research on wind is also important for the temperature on Mars.

### Wind on Mars

There are many marks left by the wind on Mars from dust devil tracks developed in seconds to kilometer-scale dune fields formed over thousands of years. Some eolian bedforms on Mars are known to be active today, as movement has been observed in time-lapse orbital images^35,36^, which can be used to infer information about the current wind regime and sediment fluxes. The formation times and spatial extent of these features cover a vast range of scales. Wind is also the key element of the seismic signals^37,38^. InSight recorded numbers of seismic events during its mission time, by which an initial conclusion was made about the influence of wind on shallow structure of the geology^39^. Much of our understanding of the phenomena is based on wind field observations. Wind field has been proved to be always challenging as it is beset by calibration problems or instrument damage^31^. The Zhurong rover has already collected and transferred wind data for about half a Martian year. We compare the wind speed and the direction of different Ls to reflect the changing pattern of the wind.

**Figure 7:**
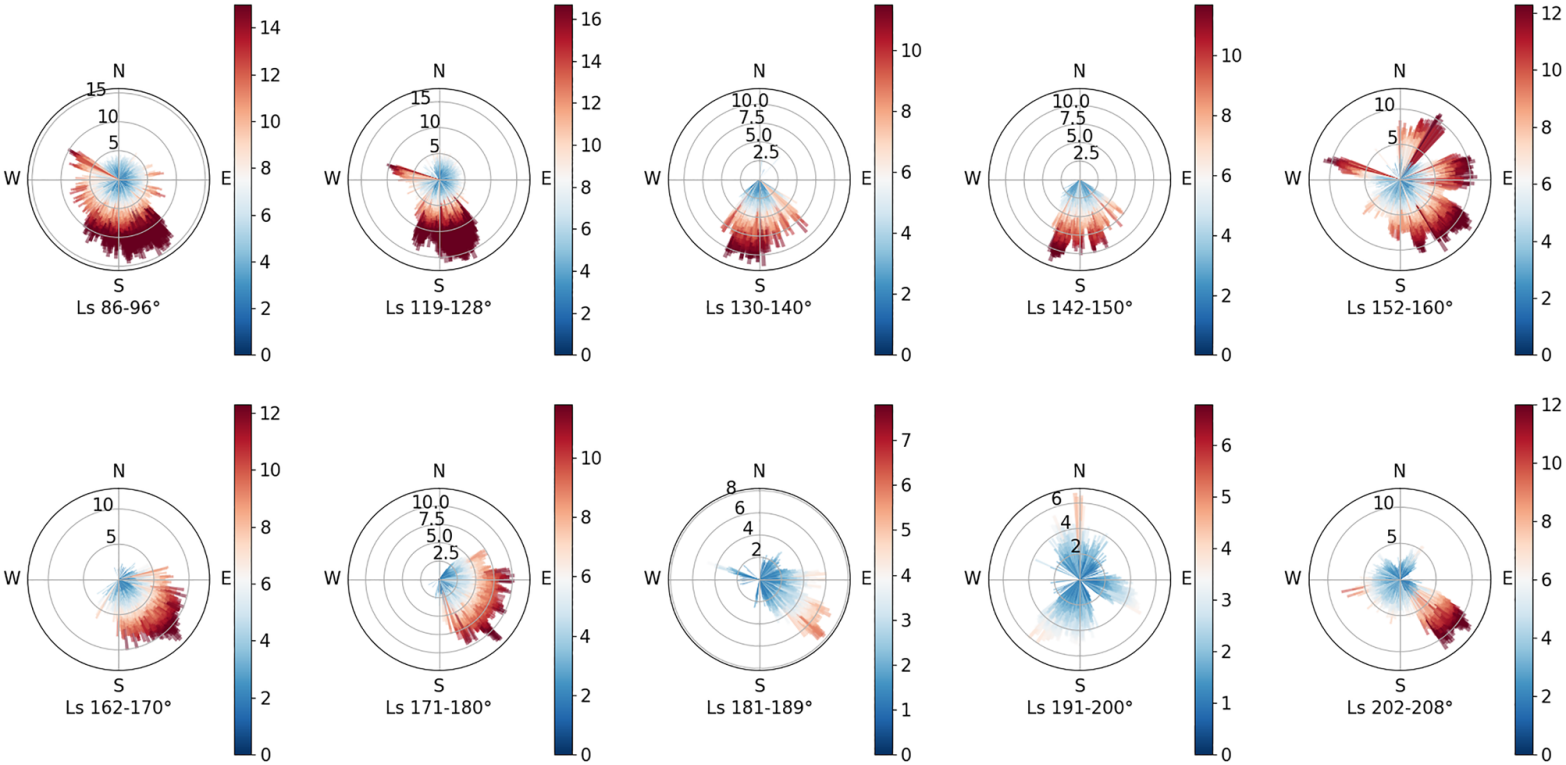
Wind speed and direction monitored by MCS during the mission time of 325 sols. Winds are analyzed by about every 10° Ls.

During the first 91 sols, the Zhurong rover works in a traditional measurement mode, which lasts until Ls = 85°. In this mode, the sensors only record the environment features for about 5 min a solar day. The recorded duration of the data is too short to reflect the true situation of the wind. As a result, it is less convincing to analyze the wind pattern by the data. The data in Fig. 7 span from Ls = 86° to Ls = 208° which are divided into 10 different Ls ranges. After Ls = 86°, MCS has shifted to a maneuvering mode in which data can be recorded for about 40 min. As the Zhurong rover works in a safe mode during the solar conjunction, there is a lack of wind data from Ls = 97° to Ls = 118°. The majority of data are recorded before LTST 12:00. As shown in Fig. 7, the wind is nearly in the true south from the northern summer solstice to Ls = 150°. After a short time of isotropic wind during Ls = 152° to Ls = 160°, the wind direction becomes southeastwards until the northern fall equinox. After that, the wind becomes slightly weak while at the end of the mission time, the wind speed becomes stronger again and turns southeast.

We find that the strongest wind recorded by Tianwen-1’s wind sensors appears in the range of Ls = 119–128°. The maximum wind speed is 16.7 m/s. According to data recorded by the digital elevation model near the landing site, there is a correlation between the shape of the crescentic bedforms^40^ and the strong south wind. As the wind is weak and isotropic since the northern fall equinox, the temperature that bumps during this period has no correlation to dust storms.

## Discussion

Zhurong has completed its first half a Martian year in the south of Utopia Planitia. These observations are the first in situ climate measurements in this region. MCS data have provided direct evidence for temperature, pressure and time-varying wind fields on the surface of Mars, offering exciting new directions. Pressure has an incontestable seasonal trend during the mission. To be more specific, it reaches a peak at the beginning of the observation, while it hits the lowest at Ls =  ~ 150°. There is generally strong agreement on the pressure and the data recorded by the previous Insight mission. As pressure shows a seasonal variation that is cyclic over the Mars year, a trigonometric function is used to fit the pressure and the solar longitude. Unlike pressure, temperature sees more chaotic changes during the sample time. Though the limitation of the sampling time and sampling range contributes to the irregular temperature changes, change patterns of temperature also show strong similarities to those of the previous missions and the modern atmospheric models. From the analysis of the wind field, a seasonal wind direction change is concluded that the south wind dominates in northern spring and summer’s morning most of the time. The wind is strong near the northern summer solstice and there is a correlation between wind and the form of crescentic bedforms.

As of today, the Zhurong rover is still on its ongoing mission to collect environment data and provide the observed details continuously. This inevitably broadens our Martian knowledge and helps to discover more possibilities for human exploration. We look forward to sustained high-quality observations by Zhurong, via which more scientific visions will be accomplished down the line.

## Methods

### Choosing of Tianwen-1’s landing site

The Tianwen-1’s landing site must meet two basic requirements: high factor of landing safety and scientific research value. Landing at lower altitudes empowers the spacecraft a longer aerodynamic deceleration time, contributing to a safer landing. Non-volcanic, flat lowlands characterize the northern hemisphere of Mars, while highlands span over the southern hemisphere. Since the Zhurong rover is powered by solar energy, the landing site lies in the low latitudes of the Martian northern hemisphere. In addition, places that have not been visited by previous missions stand at the top of landing spot list.

### Description of scientific payloads and data format

To complete the scientific objectives of “orbiting, landing, and patrolling”, the orbiter and the rover are equipped with 13 instruments to collect the data from Mars: 7 on the orbiter and 6 on the rover respectively^11^. Different data products including radar echo data, spectrum data, image data, energy spectrum data, magnetic field data, meteorological data, and acoustic data are delivered to the community where scientists can download for analysis^41^.

The data level definitions of Tianwen-1 have the same principle as PDS standards for formatting and labelling files. All data are stored as tables of N-columns by M-rows by ASCII code in time ordered. The columns are separated and of fixed sizes. Each data file comprises one solar day (hereinafter referred to as sol) of data; therefore, the number of rows in the data products is equivalent to the number of records contained in one sol. The geometric information is given by solar incidence angle and solar azimuth angle. The data file was transferred to the earth by WIFI via the relay orbiter.

### Pressure measurement

The pressure measurement is carried on by the pressure sensor array which is integrated into the instrument control unit located in the rover cabin. A gas filtering head designed to protect the pressure sensor from Martian dust is installed on the outer plate of the rover with a gas tube. The sensor element is connected to the atmosphere through the stainless gas tube and the gas filtering head. This pressure measurement scheme has been designed to provide high reliability in the harsh environment on the surface of Mars. The pressure sensor array incorporates three MEMS pressure sensors, a redundancy that can further improve the reliability of the pressure data. The sampling rate of pressure is 1 Hz and the measurement ranges from 1 Pa to 20 hPa with an accuracy of ± 7 Pa^19^.

### Air temperature measurement

The air temperature collected by Tianwen-1 mainly comes from the air temperature sensor arrays which consists of three PT1000 platinum resistance thermometers as the temperature sensitive components. The three sensors are located at the top (temperature1), middle (temperature2), and bottom (temperature3) of a PCB bracket. The air temperature and pressure sensors, about 0.5–0.6 m above the ground, are under the top board of rover’s cabin. The temperature is measured with a continuous sampling rate of 1 Hz and with an accuracy of about 0.087 K for a recorded resolution of 0.1 K. We use *T*_1_, *T*_2_ and *T*_3_ to represent the temperatures of the three sensors. The surrounding air temperature can be calculated by the method in literature 19.

### Wind measurement

MCS monitors the wind and the direction continuously, offering an opportunity to find out some evidence for surface activity from a wind-induced effect. The observations of wind field help to better understand the global and local wind regimes and their seasonal variability on Mars. Thermal film measurement principle is adopted for the wind measurement. The wind sensors, about 1.6–1.7 m above the ground, are at the top of the rover’s mast. The sensors acquire wind and direction data at a high frequency of 1 Hz and an accuracy of ~ 1.1 m/s for wind speed and ~ 6.8 degrees for wind direction^19^.

### Mars seasons and calendars

Martian axis of rotation is tilted 25.1° with respect to the plane of its orbit around the Sun. The number is similar to that of the earth, which has an axial tilt of 23.4°. In this case, this leads to the four distinct seasons on Mars like Earth. The Mars–Sun angle, named the solar longitude Ls, is used to indicate seasons on Mars: 0° corresponds to northern spring equinox, 90° to northern summer solstice (aphelion season), 180° to northern fall equinox and 270° to northern winter solstice (perihelion season). A Martian year starts at Ls 0° and spans about 1.9 Earth years, or 668.59 sols (1 sol = 88,775 s). Enumeration of Martian years and seasons is described in detail by Piqueux^42^.

### Data analysis

The first group of data was received from the MCS on Zhurong rover on 21 May, 2021, which corresponds to Ls = 49.6°. We define this moment as sol 0. The last set of data received from the MCS before the completion of manuscript, was on 12 April, 2022, corresponding to Ls 207.5°. The data we apply in this study spans over about one Earth year, namely half a Martian year, which is sufficient to explain phenomena on the surface of Mars. As of 25 May, 2022, Zhurong rover has travelled 1921 m to the suspected coastline of an ancient sea which lies on the southern part of Utopia Planitia. Though it is quite a long distance for the rover during its journey, it can be ignored when we intend to analyze the climate. In case of the limited available bandwidth for communication, full high-frequency measurements cannot be retrieved on Earth. The actual time of observation for MCS depends on availability of power, data volume, and many other issues. During the first 80 sols, MCS worked in a traditional mode, under which the sensors monitored the data only 5 min in 1 solar day for technical tests of the instrument and connection of the communication link. After the first 80 sols, MCS converted into a maneuver mode. In this scheme, sensors recorded the climate data for a longer time. It is worth mentioning that MCS does not work every day as different sensors may coordinate their working hours in case of time conflict. The running timetable of MCS resorts to its flight control conditions. A notable phenomenon was the solar conjunction which last from the late September to mid-October. During this period, Earth and Mars were on opposite sides of the Sun, which indicated that the three could be almost in a straight line. As a result, the probe entered the solar conjunction. In the process of the transit, the ground-space communication was cut off due to electromagnetic radiation interference, forcing the orbiter and the rover into a safety mode and halt their probe work. After this period, the orbiter and the rover would be woken up again to resume their operations.

## Data Availability

The Tianwen-1 data used in this work was processed and produced by “Ground Research and Application System (GRAS) of China’s Lunar and Planetary Exploration Program MCS, DEM, DIM Dataset, it can be downloaded at https://clpds.bao.ac.cn/web/zhmanager/mars1.
